# Intimate partner violence perpetration, standard and gendered STI/HIV risk behaviour, and STI/HIV diagnosis among a clinic-based sample of men

**DOI:** 10.1136/sti.2009.036368

**Published:** 2009-07-21

**Authors:** M R Decker, G R Seage, D Hemenway, J Gupta, A Raj, J G Silverman

**Affiliations:** 1 Harvard School of Public Health, Department of Society, Human Development and Health, Boston, Massachusetts, USA; 2 Harvard School of Public Health, Department of Epidemiology, Boston, Massachusetts, USA; 3 Harvard School of Public Health, Department of Health Policy and Management, Boston, Massachusetts, USA; 4 Yale University Center for Interdisciplinary Research on AIDS, New Haven, Connecticut, USA; 5 Boston University School of Public Health, Department of Social and Behavioral Sciences, Boston, Massachusetts, USA

## Abstract

**Background:**

The estimated one in three women worldwide victimised by intimate partner violence (IPV) consistently demonstrate elevated STI/HIV prevalence, with their abusive male partners’ risky sexual behaviours and subsequent infection increasingly implicated. To date, little empirical data exist to characterise the nature of men’s sexual risk as it relates to both their violence perpetration, and STI/HIV infection.

**Methods:**

Data from a cross-sectional survey of men ages 18–35 recruited from three community-based health clinics in an urban metropolitan area of the northeastern US (n = 1585) were analysed to estimate the prevalence of IPV perpetration and associations of such violent behaviour with both standard (eg, anal sex, injection drug use) and gendered (eg, coercive condom practices, sexual infidelity, transactional sex with a female partner) forms of sexual-risk behaviour, and self-reported STI/HIV diagnosis.

**Results:**

Approximately one-third of participants (32.7%) reported perpetrating physical or sexual violence against a female intimate partner in their lifetime; one in eight (12.4%) participants self-reported a history of STI/HIV diagnosis. Men’s IPV perpetration was associated with both standard and gendered STI/HIV risk behaviours, and to STI/HIV diagnosis (OR 4.85, 95% CI 3.54 to 6.66). The association of men’s IPV perpetration with STI/HIV diagnosis was partially attenuated (adjusted odds ratio (AOR) 2.55, 95% CI 1.77 to 3.67) in the multivariate model, and a subset of gendered sexual-risk behaviours were found to be independently associated with STI/HIV diagnosis—for example, coercive condom practices (AOR 1.67, 95% CI 1.04 to 2.69), sexual infidelity (AOR 2.46, 95% CI 1.65 to 3.68), and transactional sex with a female partner (AOR 2.03, 95% CI 1.36 to 3.04).

**Conclusions:**

Men’s perpetration of physical and sexual violence against intimate partners is common among this population. Abusive men are at increased risk for STI/HIV, with gendered forms of sexual-risk behaviour partially responsible for this association. Thus, such men likely pose an elevated infection risk to their female partners. Findings indicate the need for interwoven sexual health promotion and violence prevention efforts targeted to men; critical to such efforts may be reduction in gendered sexual-risk behaviours and modification of norms of masculinity that likely promote both sexual risk and violence

STI/HIV is rapidly spreading among young urban men and women in the USA, with an estimated half of all new infections occurring among those ages 15–24 years.^1^ Evidence from the USA and elsewhere illustrates women’s elevated STI/HIV risk based on their intimate partner victimisation (IPV),^2^ ^3^ ^4^ ^5^ ^6^ with abusive male partners’ sexual-risk behaviour^7^ ^8^ ^9^ ^10^ ^11^ ^12^ ^13^ ^14^ ^15^ ^16^ ^17^ and subsequent STI/HIV infection^8^ ^9^ ^15^ ^17^ increasingly considered responsible for this association.

As IPV cannot cause STI/HIV in the absence of pathogen exposure, the body of research devoted to understanding elevated STI/HIV among male IPV perpetrators has largely focused on articulating patterns of STI/HIV risk behaviour among this population. Standard STI/HIV risk behaviours, that is, those commonly assessed within surveillance efforts^18^ ^19^ and recommended for behavioural interventions,^20^ ^21^ ^22^ (eg, multiple sex partnering, inconsistent condom use, injection drug use and anal sex) are consistently found to be more prevalent among IPV perpetrators.^10^ ^11^ ^12^ ^13^ ^14^ ^15^ ^16^ ^17^ These data suggest that male perpetrators of violence are more likely to contract and transmit STI/HIV infection based on such behaviour.

A growing body of research suggests that men’s abuse of female partners reflects gender-based power imbalances which may extend to gendered forms of sexual-risk behaviour.^10^ ^23^ While the standardly assessed STI/HIV risk behaviours described above may reflect gender-based power imbalances, recent attention to the gendered nature of STI/HIV risk^24^ ^25^ ^26^ has prompted increased recognition of sexual-risk behaviours that are explicitly rooted in gender-based power imbalances and, importantly, may also represent additional dimensions of infection risk. For example, coercive sexual negotiation (eg, women’s limited ability to refuse sex or insist on condom use in the face of violence) is common in the context of abuse;^14^ ^27^ ^28^ ^29^ ^30^ consequent unwanted and unprotected sex may prompt physical trauma (ie, tearing or lacerations).^26^ ^31^ Thus, coercive sexual negotiation may heighten the risk for sexual transmission from an infected partner. IPV perpetrators are also more likely to engage in transactional sex (ie, engaging in sex with women in prostitution^7^ ^10^ ^16^) as well as other materially or financially motivated sexual encounters;^7^ such behaviour is considered to both manifest gendered power and control^7^ and, importantly, represent higher-risk sexual contact with respect to STI/HIV.^32^ ^33^ ^34^ Extramarital partnering and other forms of sexual infidelity (ie, concurrent undisclosed sexual partnerships) similarly pose both additional STI/HIV risk^35^ and relate to men’s IPV perpetration.^8^ ^9^ Such infidelity likewise appears rooted in gender-based power imbalances and culturally sanctioned norms of masculinity.^36^ ^37^ Taken together, these data illustrate that, in addition to standard STI/HIV risk behaviours found to be more prevalent among abusive men, gendered sexual-risk behaviours (ie, related to men’s entitlement to sexual power, control and access) may represent additional and distinct infection risk and, perhaps, partially explain their increased likelihood of STI/HIV acquisition and subsequent transmission.

However, previous studies have not simultaneously considered standard and gendered STI/HIV risk behaviours. Such designs are necessary for determination of which forms of risk are most relevant in explaining the elevated prevalence of STI/HIV among abusive men and, by extension, mechanisms by which abusive men may be most likely to become infected (ie, whether IPV perpetration may be a marker for abusive men’s greater likelihood to engage in standard forms of STI/HIV risk behaviour, and/or whether abuse may reflect greater engagement in gendered, and qualitatively different, forms of sexual risk which in turn may pose risk for STI/HIV acquisition and transmission).

The current study is designed to estimate associations of (1) IPV perpetration with both standard (eg, injection drug use, anal sex, multiple partnering) and gendered (eg, coercive condom negotiation practices, sexual infidelity, sex purchasing) forms of STI/HIV risk behaviour, (2) IPV perpetration and both standard and gendered STI/HIV risk behaviours with STI/HIV diagnosis and (3) IPV perpetration with STI/HIV diagnosis while controlling for standard and gendered STI/HIV risk behaviours among a clinic-based sample of young urban men.

## Methods

### Design and setting

The current study utilises data collected between January 2005 and December 2006 via a cross-sectional survey conducted in collaboration with three urban community health centers (CHC). At each CHC, all English-, Spanish- or Portuguese-speaking men presenting to the main reception desk were screened for eligibility (ages 18–35 years) by trained research staff fluent in these languages. As this investigation was originally designed to evaluate risk and protective factors for IPV perpetration, the age range 18–35 years was selected to maximise statistical power, as this age group is considered at greatest risk for such perpetration.^38^ Those meeting eligibility criteria and expressing interest in the study were escorted to a private area of the CHC where verbal consent was obtained to preserve participant anonymity. Following informed consent procedures, participants completed a survey using Audio Computer-Assisted Survey Instrument (ACASI), a computer-based survey tool in which participants self-administer the survey while questions and answer choices are read aloud to them over headphones to reduce potential literacy barriers. ACASI has been demonstrated effective in increasing reporting of sensitive behaviours^39^ and has been recommended specifically for research concerning violence perpetration.^40^ Following completion of the 30 min survey, participants received a $20 prepaid debit card and a list of local community resources for violence prevention, health promotion and mental health services. All study materials were available in English, Spanish and Portuguese. Of the 3430 men approached for the study, 2229 agreed to participate (65%). Given the need to obtain verbal consent prior to data collection, no data were obtained on non-participants. The primary reason for non-participation was lack of time. Of the 2229 participants, a small number were considered non-responsive based on extensive missing data (n = 75); of the remaining 2154 participants, 1711 men (79%) indicated ever having had sexual intercourse; 1585 (93%) provided complete data concerning the predictor and outcome variables; present analyses are limited to these 1585 participants. The youngest participants (men aged 18–21 years) were slightly more likely to fail to provide adequate data for analyses; no other evidence of bias was detected.

### Measures

All measures were self-reported. Lifetime history of IPV perpetration against female partners was assessed via 14 items modified from the Conflict Tactics Scale 2 (CTS-2)^41^ and the Sexual Experiences Survey (SES).^42^ Six items assessed history of physical violence perpetration, and eight items assessed history of sexual violence perpetration against a female intimate partner (ie, a current or former dating or marital partner). Based on these assessments, a single dichotomous variable was created to reflect lifetime history of IPV perpetration such that report of any physical (eg, pushed, punched or shoved) or sexual (eg, insisted on sexual activity, used force for sexual activity) violence against an intimate partner indicated a lifetime history of IPV with those reporting no to all items serving as the referent group. Lifetime history of STI/HIV diagnosis was assessed via the single item, “Have you ever been told by a medical professional that you have a sexually transmitted disease (Chlamydia, herpes, gonorrhea, HIV, genital warts)?” Single items were also used to assess covariates of interest, specifically demographics (age, race/ethnicity and education level), standard STI/HIV risk factors (lifetime history of injection drug use, lifetime history of anal sex and past-year history of multiple sexual partners), and gendered STI/HIV risk factors specific to sexual encounters with female partners, specifically lifetime history of transactional sex, that is, “Have you ever traded drugs, money or a place to stay in exchange for sex from a girl or woman?,” lifetime history of anger in response to condom request, that is, “Have you ever gotten mad at a girl/woman for asking to use a condom when you have sex?,” lifetime history of coerced condom non-use, that is, “Have you ever made a girl/woman have sex without a condom even though she wanted to use one?,” lifetime history of sexual infidelity which serves as a proxy for concurrent sexual partnerships, that is, “Have you ever had sex with some other girl/woman when you were supposed to only be having sex with one girlfriend or wife of yours?”

### Analysis

Prevalence estimates were calculated for lifetime IPV perpetration for the total sample and by demographic factors; differences in IPV perpetration based on these factors were assessed via χ^2^ analyses; significance for all analyses was set at p<0.05. Prevalence estimates for standard and gendered STI/HIV risk behaviours were calculated for the sample and based on male IPV perpetration. Logistic regression models were constructed to estimate odds ratios for each risk behaviour based on IPV perpetration, adjusted for all potential demographic confounders (ie, age, education and relationship status) and location of recruitment.

A final series of analyses considered the standard and gendered STI/HIV risk behaviours as exposures in order to determine their associations with STI/HIV diagnosis. Prevalence estimates of lifetime STI/HIV diagnosis were calculated for the total sample and by IPV perpetration and standard and gendered STI/HIV risk behaviours. Logistic regression models were constructed to estimate crude odds ratios (ORs) and 95% CIs of lifetime STI/HIV diagnosis based on the main exposure (IPV perpetration) and both standard and gendered STI/HIV risk factors. Finally, in order to evaluate the which standard and gendered STI/HIV risk behaviours may be responsible for associations of IPV perpetration with STI/HIV diagnosis, a multivariate model was constructed to consider all exposures, that is, IPV perpetration, and standard and gendered STI/HIV behaviours. The multivariate model was adjusted for all potential demographic confounders (ie, age, education and relationship status) and location of recruitment. The Harvard School of Public Health Human Subjects Committee approved all study procedures.

## Results

### Intimate partner violence perpetration

Approximately one in three (32.7%) participants reported a lifetime history of physical or sexual violence perpetration against a female partner (table 1). The prevalence of IPV perpetration varied across age groups, with men aged 22–25 years most likely to perpetrate (38.2%; p = 0.001).

**Table 1:** Sample demographics and associations with lifetime history of intimate partner violence (IPV) perpetration and STI/HIV diagnosis (n = 1585)

	Sample (%)*	Lifetime history of intimate partner violence perpetration (%)†	χ^2^ p value	Odds ratio (95% CI)	Lifetime history of STI/HIV diagnosis (%)†	χ^2^ p value	Odds ratio (95% confidence interval)
Total	100	32.7			12.4		
Age (years)			0.001			<0.001	
18–20	24.1	25.9		Reference	5.8		Reference
21–25	27.1	35.5		1.52 (1.12 to 2.04)	10.1		1.64 (0.98 to 2.75)
26–30	21.4	31.5		1.27 (0.92 to 1.75)	11.9		1.97 (1.17 to 2.33)
31–35	27.4	36.7		1.60 (1.19 to 2.15)	20.7		3.81 (2.39 to 6.08)
Race/ethnicity			0.032			0.018	
Non-Hispanic White	8.2	33.3		0.90 (0.60 to 1.33)	8.5		0.53 (0.28 to 1.01)
Non-Hispanic Black	48.6	35.9		Reference	15.0		Reference
Hispanic	31.9	30.4		0.78 (0.61 to 0.99)	10.9		0.69 (0.49 to 0.98)
Other	11.3	25.7		0.62 (0.43 to 0.89)	8.4		0.52 (0.30 to 0.91)
Highest educational attainment			0.762			0.348	
<High school education	27.9	32.5		1.05 (0.79 to 1.39)	11.6		0.80 (0.54 to 1.18)
High school or general education development	44.4	33.7		1.11 (0.86 to 1.43)	11.9		0.82 (0.57 to 1.16)
Some college or beyond	27.7	31.7		Reference	14.5		Reference

*Column per cent.

†Row per cent.

IPV perpetration also varied across racial/ethic groupings, with non-Hispanic Black men reporting the highest levels of perpetration (35.9%), followed by non-Hispanic White men (33.3%; p = 0.032). No differences were detected based on education.

### Intimate partner violence perpetration and standard and gendered STI/HIV Risk Behaviour

Both standard and gendered STI/HIV risk factors were more prevalent among IPV perpetrators as compared with their non-abusive counterparts (table 2). The most commonly reported standard STI/HIV risk behaviour was anal sex (45.6%), with IPV perpetrators over twice as likely to engage in such behaviour (58.8% vs 39.2%; adjusted odds ratio (AOR) 2.12, 95% CI 1.70 to 2.64). The most prevalent gendered STI/HIV risk behaviour was sexual infidelity, that is, cheating on a main partner (48.1%); IPV perpetrators demonstrated over three times the odds of engaging in such behaviour (AOR 3.91, 95% CI 3.10 to 4.91). Coercive condom practices in the forms of condom refusal and anger in response to condom request were common, and were more prevalent among IPV perpetrators relative to non-abusers (30.8% vs 10.4%; AOR 3.71, 95% CI 2.82 to 4.88; 22.5% vs 5.6%, AOR 4.88, 95% CI 3.47 to 6.85, respectively).

**Table 2:** Prevalence of standard and gendered STI/HIV risk behaviour and associations with men’s IPV (intimate partner violence) perpetration (n = 1585)

	Sample (%)*	Percentage* among IPV perpetrators n = 519	Percentage* among non-IPV perpetrators n = 1066	Adjusted odds ratio† (95% confidence interval)
Standard STI/HIV risk behaviour				
Sex partners in past 12 months >6	19.9	25.4	17.2	1.72 (1.33 to 2.23)
Lifetime history of anal sex	45.6	58.8	39.2	2.12 (1.70 to 2.64)
Lifetime history of injection drug use	7.6	13.1	4.9	2.58 (1.75 to 3.81)
Gendered STI/HIV risk behaviour				
Lifetime history of sexual infidelity/concurrent partnerships	48.1	69.9	37.4	3.91 (3.10 to 4.91)
Lifetime history of coerced condom non-use (condom refusal)	17.1	30.8	10.4	3.71 (2.82 to 4.88)
Lifetime history of anger in response to condom request	11.2	22.5	5.6	4.88 (3.47 to 6.85)
Lifetime history of transactional sex with female partners	13.5	29.1	5.9	6.22 (4.50 to 8.61)

*Column per cent.

†Adjusted for age, race/ethnicity, recruitment site.

### STI/HIV diagnosis

STI/HIV diagnosis across the lifetime was reported by 12.4% of men (table 3). IPV perpetration was bivariately associated with lifetime STI/HIV diagnosis, with approximately one-quarter of abusive men (24.9%) experiencing STI/HIV as compared with only 6.4% of non-abusive men. IPV perpetration as well as all forms of standard and gendered STI/HIV risk behaviour were associated with STI/HIV diagnosis in bivariate analyses. While partially attenuated, the association of IPV with STI/HIV diagnosis persisted in the multivariate model (AOR 2.55, 95% CI 1.77 to 3.67). Additional factors significantly associated with lifetime STI/HIV diagnosis in the multivariate model were gendered STI/HIV risk behaviours only, specifically having been sexually unfaithful (AOR 2.46, 95% CI 1.65 to 3.68), anger in response to condom request (AOR 1.67, 95% CI 1.04 to 2.69) and transactional sex (AOR 2.03, 95% CI 1.36 to 3.04).

**Table 3:** Prevalence of STI/HIV diagnosis and associations with men’s intimate partner violence perpetration and STI/HIV risk behaviour (n = 1585)

	Lifetime history of STI/HIV diagnosis		
	Percentage*	Odds ratio (95% confidence interval)	Adjusted odds ratio† (95% confidence interval)
Lifetime intimate partner violence perpetration			
No	6.4	Reference	Reference
Yes	24.9	4.85 (3.54 to 6.66)	2.55 (1.77 to 3.67)
Standard STI/HIV risk behaviour			
Sex partners in past 12 months			
< 6	11.0	Reference	Reference
⩾6	18.1	1.78 (1.27 to 2.50)	1.30 (0.88 to 1.92)
Lifetime history of anal sex			
No	7.7	Reference	Reference
Yes	18.0	2.60 (1.90 to 3.56)	1.41 (0.99 to 2.01)
Lifetime history of injection drug use			
No	11.1	Reference	Reference
Yes	28.3	3.16 (2.06 to 4.85)	1.30 (0.77 to 2.17)
Gendered STI/HIV risk behaviour with female partners			
Lifetime history of sexual infidelity/concurrent partnerships			
No	5.4	Reference	Reference
Yes	20.1	4.45 (3.13 to 6.32)	2.46 (1.65 to 3.68)
Lifetime history of coerced condom non-use (condom refusal)			
No	10.0	Reference	Reference
Yes	24.4	2.91 (2.09 to 4.05)	0.89 (0.57 to 1.38)
Lifetime history of anger in response to condom request			
No	9.9	Reference	Reference
Yes	32.8	4.45 (3.11 to 6.37)	1.67 (1.04 to 2.69)
Lifetime history of transactional sex with female partners			
No	8.7	Reference	Reference
Yes	36.5	6.03 (4.31 to 8.44)	2.03 (1.36 to 3.04)

*Row per cent.

†Adjusted for age, education, race/ethnicity, recruitment site and all covariates in the table.

## Discussion

Findings indicate that, relative to their non-abusive counterparts, men who perpetrate violence against female partners engage in higher levels of both standard and gendered STI/HIV risk behaviour, and demonstrate an elevated prevalence of STI/HIV. Moreover, it appears that the elevated STI/HIV prevalence observed among abusive men may be better explained by gendered forms of STI/HIV risk behaviour (eg, sexual infidelity, coercive condom practices and involvement in transactional sex with a female partner) than standardly assessed risk behaviours. Given that almost one-third of participants reported perpetrating physical or sexual violence against a female partner, identified associations of IPV perpetration with gendered forms of STI/HIV risk and subsequent STI/HIV acquisition strongly indicate that men who perpetrate partner violence should be prioritised for intervention efforts as they likely, in addition to causing a broad range of physical and psychological injuries, pose heightened STI/HIV risk to their female partners.

Our findings of elevated sexual risk and STI/HIV diagnosis among male perpetrators of violence are consistent with prior research.^8^ ^9^ ^10^ ^14^ ^15^ ^16^ ^43^ Advancing this body of knowledge, current evidence indicates that the association of men’s IPV perpetration with their STI/HIV may be better explained by gendered than standard STI/HIV risk sources. While both standard (ie, anal sex, injection drug use) and gendered STI/HIV risk behaviours (ie, transactional sex, coercive condom practices, sexual infidelity) were found associated with both IPV perpetration and STI/HIV diagnosis, only gendered forms of sexual risk were independently associated with STI/HIV in the final multivariate model. Current findings advance our understanding of potential mechanisms underpinning findings of elevated STI/HIV among abusive men and suggest that the gendered, and potentially qualitatively riskier, sexual-risk behaviours more common among abusive men may contribute to their elevated STI/HIV infection.

Surprisingly, IPV perpetration remained significantly associated with STI/HIV diagnosis in the multivariate model adjusted for both standard and gendered risk behaviours. Its persistence suggests that, while IPV perpetration in itself cannot cause STI/HIV, men’s abusive behaviour constitutes a marker for risky sexual practices above and beyond those currently captured (eg, forced unprotected sex) and, notably, beyond those traditionally assessed in STI/HIV prevention efforts (eg, injection drug use, multiple partnering). The demonstrated inability of standardly assessed STI/HIV risk behaviours to explain abusive men’s elevated STI/HIV infection suggests that modification of these behaviours via traditional prevention strategies may be insufficient to reduce STI/HIV among IPV perpetrators and their female partners.

This finding also indicates that the range of STI/HIV risk behaviour assessed within the current study, while more detailed than standard surveillance methods,^18^ ^19^ ^44^ was still inadequate in scope and/or precision; factors not fully captured warrant further consideration, including coital frequency, context and nature of men’s anal sex (eg, receptive or insertive), and forced and unprotected sex in the contexts of both substance use^45^ and casual concurrent partnerships.^14^ Although female-to-male STI/HIV transmission is relatively inefficient biologically,^46^ and recent evidence indicates that abusive men are more likely than their non-abusive counterparts to acquire HIV outside the marital relationship,^17^ men may have acquired STI/HIV from their female partners, particularly if such women have been infected in a prior abusive relationship. Thus, consideration of female partner STI/HIV status will improve the clarity of future investigations. As IPV perpetration cannot directly cause STI/HIV, more comprehensive assessment of gendered sexual-risk behaviours should be a priority of future research. If gendered sexual risk does, indeed, account for much of the association of IPV with STI, inclusion of such improved assessment in models predicting STI/HIV will result in greater attenuation of the association of IPV with STI/HIV. In other words, if IPV is a marker for other gendered sexual-risk behaviours, the more comprehensive assessment of such behaviours will attenuate the role of IPV to a greater extent in predicting STI/HIV. Additional prospective investigation using couples as the unit of analysis is recommended to clarify the mechanisms and temporal sequencing of IPV perpetration, sexual risk and STI acquisition.

As has been posited in other contexts facing similar patterns of elevated STI/HIV risk among abusive men,^10^ ^17^ ^24^ men’s violence perpetration against female partners and gendered sexual risk may stem from a common source. Qualitative and quantitative evidence indicates that men’s endorsement of masculinity norms which support men’s entitlement to sexual control of women and adversarial sexual beliefs relate to both IPV perpetration^23^ ^47^ ^48^ and sexual-risk behaviour.^7^ ^23^ ^45^ ^48^ Such individual and socially reinforced norms may explain the observed clustering of STI/HIV risk and diagnosis with IPV perpetration,^10^ ^24^ and, as such, serve as a basis for further research and programmatic efforts to address these inter-related threats.

The current findings should be considered in light of several limitations in addition to those previously mentioned. Cross-sectional analysis precludes conclusions regarding temporality; prospective work is needed to determine the relative sequencing and impact of these STI/HIV risk behaviours and IPV on men’s STI/HIV acquisition and subsequent transmission. Several sampling issues should be considered, including the 65% response rate and the inability to study potential biases among non-respondents due to ethical considerations. Additionally, the prevalence of sexual intercourse was lower than anticipated for this adult sample and may reflect under-reporting possibly based on confusion regarding the terminology “sexual intercourse,” as no definition was provided. While the sample was limited to those men reporting sexual activity, STI/HIV can also be transmitted without penetrative sex. Younger men were slightly more likely to fail to provide complete data on the exposures and outcomes of interest. While these factors limit the generalizability of findings, the nature and direction of any potential bias introduced remain unclear. All data are self-reported, rendering them subject to potential inaccuracies attributable to social desirability, recall bias, intentional distortions or non-candid responses.^49^ Use of ACASI likely minimised these threats, given the demonstrated ability of ACASI to enhance the quality of assessment of sensitive behaviours.^39^ Reliance on self-reported STI/HIV diagnosis likely underestimates the number of individuals infected.^50^ Future studies including biological assessment of STI/HIV as well as more comprehensive and specific sexual-risk assessments (eg, condom non-use in the context of multiple partnering, coital frequency, forced unprotected sex) may clarify the current findings. Given the use of a single urban metropolitan area, with high representation from young men of colour, findings may not generalise to broader populations of men. However, the current sample may be considered particularly informative, as it reflects individuals receiving care within community-based health centres, suggesting that such men may be readily accessed for intervention in this medical setting. Moreover, the elevated STI/HIV risk demonstrated among this population^1^ ^44^ ^51^ ^52^ ^53^ highlights the relevance of the current sample for programmatic implications.

The current findings indicate a high level of IPV perpetration among young, urban, adult men attending community health centres, with approximately one in three men reporting perpetration of physical or sexual violence against an intimate partner. These abusive men’s increased risk of STI/HIV diagnosis appears to be better explained by their involvement in gendered forms of STI/HIV risk behaviour as compared with those more standardly assessed. These data bolster calls to integrate men’s violence perpetration prevention within STI/HIV prevention efforts,^24^ and indicate that such efforts should specifically target gendered STI/HIV risk behaviours. Integrated efforts should include a focus on modifying masculinity norms that support men’s entitlement to sexual control of and access to women, given evidence that such attitudes appear to underpin both IPV perpetration^23^ ^47^ ^48^ and sexual-risk behaviour.^7^ ^23^ ^45^ ^48^ Recent evidence of intervention efficacy in reducing men’s IPV perpetration, sexual-risk behaviour and STI in the South African context^54^ suggest the utility of integrated efforts; current findings indicate the need to evaluate this prevention approach in the US to stem the increasingly interwoven epidemics of men’s IPV perpetration and STI/HIV.

**Key messages**

- Approximately one in three young adult urban men reported a lifetime history of physical or sexual violence perpetration against a female intimate partner.
- Men’s abuse of their female partners was associated with elevated sexual risk as well as STI/HIV diagnosis.

## References

[1] CDC Trends in reportable sexually transmitted disease in the United States, 2005. Atlanta: Centers for Disease Control and Prevention, 2006

[2] Decker MR Silverman JG Raj A Dating violence and sexually transmitted disease/HIV testing and diagnosis among adolescent females. Pediatrics 2005;116:e272–6 16061580 10.1542/peds.2005-0194

[3] Dunkle KL Jewkes RK Brown HC Gender-based violence, relationship power, and risk of HIV infection in women attending antenatal clinics in South Africa. Lancet 2004;363:1415–21 15121402 10.1016/S0140-6736(04)16098-4

[4] Salam MA Alim MA Noguchi T Spousal abuse against women and its consequences on reproductive health: A study in the urban slums in Bangladesh. Matern Child Health J 2006;10:83–94 16362235 10.1007/s10995-005-0030-6

[5] Wingood GM DiClemente RJ Raj A Adverse consequences of intimate partner abuse among women in non-urban domestic violence shelters. Am J Prev Med 2000;19:270–5 11064231 10.1016/s0749-3797(00)00228-2

[6] Silverman JG Decker MR Saggurti N Intimate partner violence and HIV infection among married Indian women. JAMA 2008;300:703–10 18698068 10.1001/jama.300.6.703

[7] Dunkle KL Jewkes R Nduna M Transactional sex with casual and main partners among young South African men in the rural Eastern Cape: prevalence, predictors, and associations with gender-based violence. Soc Sci Med 2007;65:1235–48 17560702 10.1016/j.socscimed.2007.04.029 PMC2709788

[8] Martin SL Kilgallen B Tsui AO Sexual behaviors and reproductive health outcomes: associations with wife abuse in India. JAMA 1999;282:1967–72 10580466 10.1001/jama.282.20.1967

[9] Silverman JG Decker MR Kapur NA Violence against wives, sexual risk and sexually transmitted infection among Bangladeshi men. Sex Transm Infect 2007;83:211–15 17301104 10.1136/sti.2006.023366 PMC2659096

[10] Dunkle KL Jewkes RK Nduna M Perpetration of partner violence and HIV risk behaviour among young men in the rural Eastern Cape, South Africa. AIDS 2006;20:2107–14 17053357 10.1097/01.aids.0000247582.00826.52

[11] Eby KK Campbell JC Sullivan CM Health effects of experiences of sexual violence for women with abusive partners. Health Care Women Int 1995;16:563–76 8707690 10.1080/07399339509516210

[12] el-Bassel N Fontdevila J Gilbert L HIV risks of men in methadone maintenance treatment programs who abuse their intimate partners: a forgotten issue. J Subst Abuse 2001;13 (1-2):29–43 11547622 10.1016/s0899-3289(01)00068-2

[13] Peedicayil A Sadowski LS Jeyaseelan L Spousal physical violence against women during pregnancy. BJOG 2004;111:682–7 15198758 10.1111/j.1471-0528.2004.00151.x

[14] Raj A Santana MC La Marche A Perpetration of intimate partner violence associated with sexual risk behaviors among young adult men. Am J Public Health 2006;96:1873–8 16670216 10.2105/AJPH.2005.081554 PMC1586132

[15] Raj A Reed E Welles SL Intimate partner violence perpetration, risky sexual behavior, and STI/HIV diagnosis among heterosexual African American men. Am J Men’s Health 2008;2:291–5 19477792 10.1177/1557988308320269

[16] Gilbert L El-Bassel N Wu E Intimate partner violence and HIV risks: a longitudinal study of men on methadone. J Urban Health 2007;84:667–80 17701458 10.1007/s11524-007-9214-2 PMC2231853

[17] Decker MR Seage GR 3rd Hemenway D Intimate partner violence functions as both a risk marker and risk factor for women’s HIV infection: findings from Indian husband–wife dyads. *J Acquir Immune Defic Syndr* 2009;51:593–600 10.1097/QAI.0b013e3181a255d6 PMC3521617 19421070

[18] CDC National youth risk behavior survey. Atlanta: Centers for Disease Control and Prevention, 2007

[19] CDC Behavioral risk factor surveillance system survey questionnaire. Atlanta: Centers for Disease Control and Prevention, 2007

[20] Johnson BT Scott-Sheldon LA Smoak ND Behavioral interventions for African Americans to reduce sexual risk of HIV: a meta-analysis of randomized controlled trials. J Acquir Immune Defic Syndr 2009;51:492–501 19436218 10.1097/QAI.0b013e3181a28121 PMC3021417

[21] Kalichman S Simbayi L Cain D Heterosexual anal intercourse among community and clinical settings in Cape Town, South Africa. Sex Transm Infect 2009 10.1136/sti.2008.035287 PMC3017216 19429569

[22] Risser JM Padgett P Wolverton M Relationship between heterosexual anal sex, injection drug use and HIV infection among black men and women. Int J STD AIDS 2009;20:310–14 19386966 10.1258/ijsa.2008.008394

[23] Santana MC Raj A Decker MR Masculine gender roles associated with increased sexual risk and intimate partner violence perpetration among young adult men. J Urban Health 2006;83:575–85 16845496 10.1007/s11524-006-9061-6 PMC2430489

[24] Dunkle KL Jewkes R Effective HIV prevention requires gender-transformative work with men. Sex Transm Infect 2007;83:173–4 17569718 10.1136/sti.2007.024950 PMC2659081

[25] Martin SL Curtis S Gender-based violence and HIV/AIDS: recognising links and acting on evidence. Lancet 2004;363:1410–11 15121398 10.1016/S0140-6736(04)16133-3

[26] WHO Violence against women and HIV/AIDS: critical intersections—intimate partner violence and HIV/AIDS. Geneva: WHO, 2004

[27] Fox AM Jackson SS Hansen NB In their own voices: a qualitative study of women’s risk for intimate partner violence and HIV in South Africa. Violence Against Women 2007;13:583–602 17515407 10.1177/1077801207299209

[28] Go VF Sethulakshmi CJ Bentley ME When HIV-prevention messages and gender norms clash: The impact of domestic violence on women’s HIV risk in the slums of Chennai, India. AIDS and Behavior 2003;7:263–72 14586189 10.1023/a:1025443719490

[29] Sivaram S Johnson S Bentley ME Sexual health promotion in Chennai, India: Key role of communication among social networks. Health Promotion International 2005;20:327–33 15964884 10.1093/heapro/dai012

[30] Wingood GM DiClemente RJ The effects of an abusive primary partner on the condom use and sexual negotiation practices of African–American women. Am J Public Health 1997;87:1016–18 9224187 10.2105/ajph.87.6.1016 PMC1380941

[31] Slaughter L Brown CR Crowley S Patterns of genital injury in female sexual assault victims. Am J Obstet Gynecol 1997;176:609–16 9077615 10.1016/s0002-9378(97)70556-8

[32] Madhivanan P Hernandez A Gogate A Alcohol use by men is a risk factor for the acquisition of sexually transmitted infections and human immunodeficiency virus from female sex workers in Mumbai, India. Sex Transm Dis 2005;32:685–90 16254543 10.1097/01.olq.0000175405.36124.3b PMC3709449

[33] Rodrigues JJ Mehendale SM Shepard ME Risk factors for HIV infection in people attending clinics for sexually transmitted diseases in India. BMJ 1995;311:283–6 7633230 10.1136/bmj.311.7000.283 PMC2550353

[34] Gibney L Saquib N Metzger J Behavioral risk factors for STD/HIV transmission in Bangladesh’s trucking industry. Soc Sci Med 2003;56:1411–24 12614693 10.1016/s0277-9536(02)00138-7

[35] Manhart LE Aral SO Holmes KK Sex partner concurrency: measurement, prevalence, and correlates among urban 18–39-year-olds. Sex Transm Dis 2002;29:133–43 11875374 10.1097/00007435-200203000-00003

[36] O’Sullivan LF Hoffman S Harrison A Men, multiple sexual partners, and young adults’ sexual relationships: understanding the role of gender in the study of risk. J Urban Health 2006;83:695–708 16758335 10.1007/s11524-006-9062-5 PMC2430487

[37] Carey MP Senn TE Seward DX Urban African–American men speak out on sexual partner concurrency: findings from a qualitative study. *AIDS Behav*. In press. 10.1007/s10461-008-9406-0 PMC2815251 18483847

[38] Institute of Medicine Violence in families. Washington: National Academy Press, 1998

[39] Metzger DS Koblin B Turner C Randomized controlled trial of audio computer-assisted self-interviewing: utility and acceptability in longitudinal studies. HIVNET Vaccine Preparedness Study Protocol Team. Am J Epidemiol 2000;152:99–106 10909945 10.1093/aje/152.2.99

[40] Abbey A Lessons learned and unanswered questions about sexual assault perpetration. J Interpers Violence 2005;20:39–42 15618559 10.1177/0886260504268117

[41] Straus MA Hamby SL Boney-McCoy S The Revised Conflict Tactics Scales (CTS2). J Fam Iss 1996;17:283–316

[42] Koss MP Gidycz CA Wisniewski N The scope of rape: incidence and prevalence of sexual aggression and victimization in a national sample of higher education students. J Consult Clin Psychol 1987;55:162–70 3494755 10.1037//0022-006x.55.2.162

[43] Frye V Latka MH Wu Y Intimate partner violence perpetration against main female partners among HIV-positive male injection drug users. J Acquir Immune Defic Syndr 2007;**46**(2 Suppl):S101–9 18089979 10.1097/QAI.0b013e31815767e6

[44] Hall HI Song R Rhodes P Estimation of HIV incidence in the United States. JAMA 2008;300:520–9 18677024 10.1001/jama.300.5.520 PMC2919237

[45] Silverman JG Decker MR Reed E Social norms and beliefs regarding sexual risk and pregnancy involvement among adolescent males treated for dating violence perpetration. J Urban Health 2006;83:723–35 16845498 10.1007/s11524-006-9056-3 PMC2430488

[46] Quinn TC Overbaugh J HIV/AIDS in women: an expanding epidemic. Science 2005;308:1582–3 15947174 10.1126/science.1112489

[47] Wood K Contextualizing group rape in post-apartheid South Africa. Cult Health Sex 2005;7:303–17 16864205 10.1080/13691050500100724

[48] Wood K Jewkes R Violence, rape, and sexual coercion: everyday love in a South African township. Gend Dev 1997;5:41–6 12292615 10.1080/741922353

[49] Aday L Designing and conducting health surveys. San Francisco: Jossey-Bass, 1989

[50] Iritani BJ Ford CA Miller WC Comparison of self-reported and test-identified chlamydial infections among young adults in the United States of America. Sex Health 2006;3:245–51 17112435 10.1071/sh06040

[51] Dean HD Steele CB Satcher AJ HIV/AIDS among minority races and ethnicities in the United States, 1999–2003. J Natl Med Assoc 2005;**97**(7 Suppl):5–12S PMC2640648 16080451

[52] Espinoza L Hall HI Hardnett F Characteristics of persons with heterosexually acquired HIV infection, United States 1999–2004. Am J Public Health 2007;97:144–9 17138918 10.2105/AJPH.2005.077461 PMC1716251

[53] Jennings JM Curriero FC Celentano D Geographic identification of high gonorrhea transmission areas in Baltimore, Maryland. Am J Epidemiol 2005;161:73–80 15615917 10.1093/aje/kwi012

[54] Jewkes R Nduna M Levin J Impact of stepping stones on incidence of HIV and HSV-2 and sexual behaviour in rural South Africa: cluster randomised controlled trial. BMJ 2008;337:a506 18687720 10.1136/bmj.a506 PMC2505093

